# Effects of an 8-Week Programmed Physical Activity Intervention on Children’s Cognitive, Emotional, and Body Movement Development—A Quasi-Experimental Study of a Preschool in Taiwan

**DOI:** 10.3390/children13030319

**Published:** 2026-02-25

**Authors:** Chiung-Hui Chiu, Jia-Ying Li, Wen-Chiung Huang

**Affiliations:** 1 Department of Infant and Child Care, College of Human Development and Health, National Taipei University of Nursing and Health Sciences, Taipei 112303, Taiwan; chiuch@ntunhs.edu.tw; 2 Department of Exercise and Health Science, College of Human Development and Health, National Taipei University of Nursing and Health Sciences, Taipei 112303, Taiwan; a0968557212@gmail.com

**Keywords:** physical activity, preschool curriculum, motor development, multiage teaching, gross motor skills

## Abstract

**Highlights:**

**What are the main findings?**

- A structured, near-daily programmed physical activity intervention effectively enhanced gross motor development in preschool children.
- Physical activity programs integrating cognitive and emotional components were associated with improvements in attention, working memory, and overall emotional competence.

**What are the implications of the main findings?**

- Well-designed physical activity programs can be feasibly integrated into daily lesson plans in preschool settings with multiage classrooms.
- Incorporating contextual storytelling into physical activity programs may support the integration of cognitive and social–emotional elements, thereby promoting young children’s physical and mental health.

**Abstract:**

**Background**: This study examined the associations between participation in an 8-week programmed physical activity (PA) intervention and cognitive, emotional, and motor development in young children. **Methods**: Thirty-three children aged 4–6 years from a kindergarten in northern Taiwan were recruited through convenience sampling. A quasi-experimental design was employed. Children in the experimental group participated in an 8-week programmed PA intervention, while those in the control group engaged in routine gross motor activities. Cognitive outcomes (attention, number sense, and working memory), emotional competence (assessed using the Emotional Competency Rating Scale for Young Children), and motor development (assessed using the Preschooler Gross Motor Quality Scale) were measured before and after the intervention. Descriptive statistics were used to summarize participant characteristics, and parametric or non-parametric tests were applied as appropriate to examine within-group and between-group differences. **Results**: Following the intervention, children aged 4–5 years (mean age = 4.16 ± 0.31 years) in the experimental group showed significant improvements in attention (*p* = 0.032, d = 0.86), whereas children aged 5–6 years (mean age = 5.34 ± 0.45 years) demonstrated improvements in both attention (*p* = 0.004, d = 1.22) and working memory (*p* = 0.026, d = 0.84). Significant gains in overall gross motor development (*p* = 0.001, d = 1.65) and object manipulation (*p* = 0.042, d = 0.89) skills were observed among children aged 4–5 years in the experimental group. Improvements in selected domains of emotional competence were observed across age groups, although these findings should be interpreted with caution given the study design. **Conclusions**: The findings provide preliminary evidence that participation in structured physical activity programs may be associated with improvements in selected cognitive, emotional, and motor outcomes in young children. From an applied perspective, the results offer ecologically valid insights that may inform the design of future physical activity programs in early childhood education settings, while highlighting the need for larger-scale studies employing more rigorous methodological designs.

## 1. Introduction

Physical activity has been consistently associated with cognitive functioning in children, particularly in domains related to attention and executive processes [1]. During early childhood, executive functions are still developing, making preschool-aged children especially vulnerable to attentional lapses and the negative effects of prolonged sedentary behavior. Consequently, structured physical activity programs have been proposed as a promising approach to support early cognitive regulation and executive functioning during this critical developmental period [2,3,4,5,6]. Collectively, existing evidence suggests that improvements in cognitive and executive functioning may represent an important mechanism through which structured physical activity supports broader developmental outcomes in early childhood.

Beyond cognitive functioning, physical activity has also been linked to children’s emotional adjustment and psychosocial well-being. Previous research indicates that regular participation in physical activity is associated with reduced depressive symptoms, lower anxiety levels, and improved emotional regulation in children [7,8,9]. Early childhood represents a formative period for emotional development, during which children gradually acquire emotional awareness and regulation through daily experiences. In this context, interventions that integrate physical activity with opportunities for social interaction may play an important role in supporting socio-emotional development in preschool-aged children.

In addition to cognitive and emotional development, early childhood is a critical period for the acquisition of fundamental movement skills (FMS), including locomotor, object control, and stability skills. These foundational skills support children’s participation in more complex physical activities and are associated with long-term physical activity engagement and health outcomes [10,11]. Structured physical activity programs that emphasize varied movement experiences and skill practice may therefore play an important role in supporting motor development during the preschool years [12,13].

Although physical activity has been widely shown to benefit cognitive and emotional functioning in children, relatively few studies have examined these domains concurrently within a single, integrated intervention framework. Most existing research has focused on isolated outcomes, such as cognition or affective symptoms, rather than considering their dynamic interaction during early childhood development [14]. This limitation is particularly evident in preschool-aged populations, where evidence remains scarce regarding whether structured physical activity interventions simultaneously influence cognitive and emotional outcomes. As a result, understanding of how physical activity may support comprehensive developmental trajectories during early childhood remains constrained [15,16]. Accordingly, further integrative investigations are warranted.

In summary, existing evidence suggests that structured PA has the potential to positively influence cognitive, emotional, and motor development in young children. Nevertheless, there remains a need for integrative intervention studies that concurrently examine these interrelated developmental domains during early childhood. Therefore, the present study aimed to investigate the effects of an 8-week structured PA intervention implemented in kindergarten settings on the cognitive, emotional, and motor development of children aged 4 to 6 years.

Study Aims and Hypotheses:

Based on the theoretical and empirical evidence reviewed above, this study adopted a quasi-experimental quantitative design to examine the effects of a structured PA intervention on multiple developmental domains in preschool-aged children. The following hypotheses were proposed.

General Hypothesis:

It was hypothesized that children who participated in the structured PA intervention would demonstrate greater overall improvements in developmental outcomes compared with children in the control group.

Specific Hypotheses:

Cognitive domain: Children in the intervention group would exhibit significantly greater improvements in cognitive-related outcomes, particularly attention-related performance, than those in the control group.

Emotional domain: Children in the intervention group would show more favorable changes in emotional-related outcomes compared with children who did not receive the intervention.

Motor domain: Children who received the structured PA intervention would demonstrate greater improvements in motor performance than children in the control group.

## 2. Materials and Methods

### 2.1. Study Participants

A quasi-experimental design was employed in this study. The inclusion criteria were children aged 4–6 years who were able to participate in the scheduled physical activity sessions and assessment procedures. The exclusion criteria included children taking medication that could affect cognitive or emotional functioning, children with unresolved musculoskeletal injuries, congenital heart disease, or any medical condition deemed unsuitable for vigorous physical activity by a physician, as well as children with identified emotional, cognitive, neurological, or speech impairments. Children’s physical status, defined as their general health condition and physical readiness to participate in physical activity, was confirmed through parental reports and kindergarten health records prior to enrollment.

This study was conducted following approval from the Institutional Review Board (IRB no.: C112017). Convenience sampling was used to recruit participants from a kindergarten in northern Taiwan. At the time of recruitment, the kindergarten comprised a total of 62 children across four classes, all of whom were within the eligible age range (4–6 years). Prior to the intervention, four children did not participate due to parental refusal or failure to return signed informed consent forms. As a result, 37 children were initially recruited. Age-appropriate verbal explanations were provided to the children, and participation was based on their willingness to engage in the activities.

During the study period, four parents withdrew their children prior to post-intervention assessments, resulting in a final analytic sample of 33 children. These withdrawals were due to parental decisions unrelated to the intervention procedures or outcome measures. No adverse events were reported, and given the limited number of withdrawals, the risk of attrition bias was considered minimal.

Participants were assigned to the experimental group (*n* = 19) or control group (*n* = 14) based on their existing classroom membership rather than random allocation, reflecting organizational constraints within the kindergarten setting. Accordingly, this quasi-experimental design may be subject to selection bias and potential classroom-level effects.

Descriptive statistics for participant sex, age, height, and weight are presented in Table 1. The experimental group consisted of 19 children. Within this group, the 4–5-year-old subgroup had a mean age of 4.16 ± 0.31 years (3 boys and 6 girls), and the 5–6-year-old subgroup had a mean age of 5.34 ± 0.45 years (6 boys and 4 girls). The control group included 14 children. In the 4–5-year-old subgroup, the mean age was 4.56 ± 0.40 years (3 boys and 2 girls), while in the 5–6-year-old subgroup, the mean age was 5.38 ± 0.46 years (6 boys and 3 girls).

### 2.2. Study Procedure

During the study period, the kindergarten arranged outdoor gross physical activity time from 10:40 a.m. to 11:30 a.m. on the schedule, with each session lasting 40 min. The experimental group participated in an 8-week programmed PA intervention five times per week, lasting 30–40 min per session. Children in the control group participated in the kindergarten’s usual gross motor activity sessions, which primarily consisted of unstructured, child-directed outdoor play. During these sessions, classroom teachers prepared a safe play environment and made commonly available equipment (e.g., balls, hoops, cones) accessible for children to select independently. No planned movement sequences, contextual storytelling, instructor-led demonstrations, or organized paired or small-group activities were provided. Teachers’ roles were limited to supervision and safety monitoring, and no systematic feedback or performance guidance was given. Although both groups were scheduled within the same general time window, activities for the experimental and control groups were conducted in separate and physically distinct outdoor areas within the kindergarten to minimize potential contamination between groups. Children in the control group did not observe or participate in the structured activities delivered to the experimental group.

To reduce potential instructor-related variability, all programmed physical activity sessions were delivered by the same instructor using standardized instructional content and procedures. This approach was intended to minimize teacher effects across intervention sessions. Before the 8-week programmed PA intervention, the experimental and control groups underwent pre-tests to assess cognitive, emotional, and body motor development. After the intervention, post-tests were carried out in Week 9 to observe changes in both groups’ cognitive, emotional, and body motor development. To further reduce assessment bias, the three assessors—each specializing in cognition, emotion, and motor development—were blinded to group assignment throughout the assessment process.

**Figure 1:**
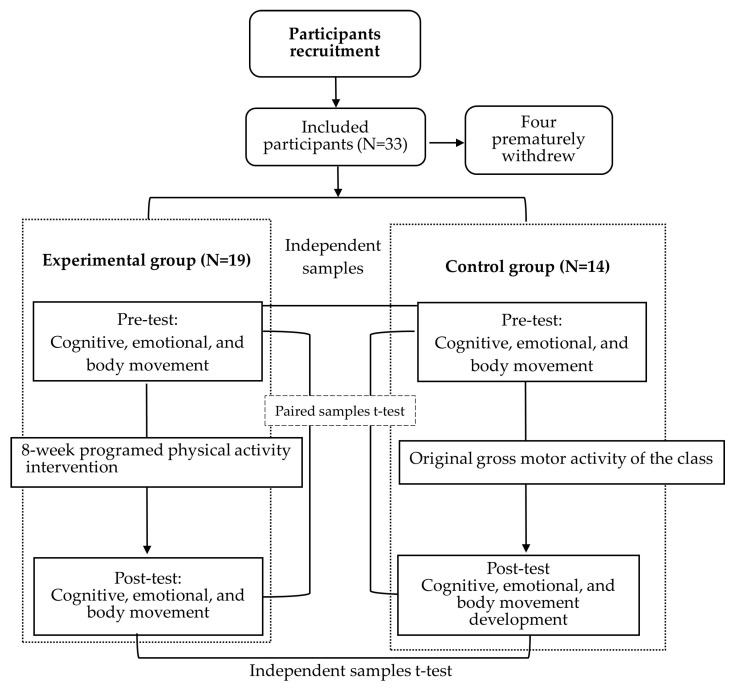
Study flowchart.

Before the start of each experimental activity in the experimental group, the procedure and safety precautions were explained to the participants. Figure 1 shows the study procedure.

### 2.3. Programmed Physical Activity Design

In this study, the items of the Preschooler Gross Motor Quality Scale (PGMQS), the experimental group’s class theme “Limb movement keeps you healthy,” and Taiwan’s Preschool Education Activity and Curriculum Guidelines were used to design the 8-week programmed physical activity (PA) intervention (Table 2), with an emphasis on fun, engagement, and safety. The intervention targeted fundamental movement skills, including stability, locomotor, and object control skills, through activities such as shooting, throwing, hitting, catching, pushing, running, jumping, and kicking.

In addition to individual motor skill practice, the program integrated contextual storytelling and paired or small-group interactive activities to promote social interaction and cognitive engagement. For example, in themed sessions such as “Soccer Prince” and “Shooting expert,” children were guided to complete movement tasks within simple narrative scenarios, requiring them to follow rules, sustain attention, and coordinate actions with peers. Activities such as “Inflatable air Stick” involved small-group cooperation to search for objects based on given clues (e.g., color or location), thereby engaging attention, working memory, and problem-solving processes while performing locomotor and object control movements. Similarly, games such as “Pushing a yoga ball” and “Bobble training” required turn-taking, shared goal achievement, and peer interaction embedded within structured motor tasks.

The activities were systematically organized to progress from simple to more complex tasks and from isolated movements to coordinated movement patterns, allowing children to gradually adapt to increasing motor and cognitive demands.

The intensity of physical activity was not objectively quantified using physiological measures (e.g., heart rate) or activity monitoring devices. Instead, activities were designed to promote active engagement through continuous movement, age-appropriate challenges, and minimal sedentary time, consistent with recommended practices for preschool physical activity. Although formal implementation fidelity measures (e.g., session checklists or observational audits) were not systematically recorded, all scheduled sessions were conducted as planned according to the intervention timetable.

### 2.4. Study Assessment

In the present study, developmental outcomes were assessed using both standardized instruments with established psychometric properties and adapted, study-specific cognitive tasks.

Standardized measures:

Emotional development was assessed using the Emotional Competency Rating Scale for Young Children (ECRS-YC) [17], and motor development was evaluated using the Preschooler Gross Motor Quality Scale (PGMQS) [18], both of which have been widely applied in early childhood research. These instruments have demonstrated acceptable reliability and validity in preschool-aged populations, as reported in their original validation studies.

Adapted cognitive tasks:

Cognitive performance was assessed across three domains: attention, number sense, and working memory. The cognitive tasks were adapted from age-appropriate measures commonly used in prior research and educational practice and were designed to be developmentally appropriate, brief, and feasible for administration in preschool classroom settings. Specifically, the attention task was adapted with reference to Weinberg (1998) [19], the number sense task was developed based on prior research on early numeracy and number sense [20], and the working memory task was adapted from previously published studies focusing on cognitive functioning in young children [21].

Prior to data collection, the content and administration procedures of the adapted cognitive tasks were reviewed by experts in early childhood education and developmental psychology to establish face validity and age appropriateness. All assessors received standardized training and followed uniform administration protocols. During testing, identical instructions were provided to all children, and no additional verbal cues, eye contact, or physical contact were permitted to minimize examiner influence. Assessors were blinded to group allocation throughout both pre- and post-intervention assessments to reduce potential assessment bias.

As these cognitive tasks were adapted for the present study, formal psychometric indices (e.g., reliability coefficients) were not established. Accordingly, findings derived from these cognitive measures are intended to capture short-term, context-specific changes in classroom-relevant cognitive behaviors and should be interpreted as exploratory rather than as definitive indicators of cognitive development.

### 2.5. Data Analysis and Processing

Data were collected using standardized assessment tools administered before and after the 8-week programmed physical activity intervention in both the experimental and control groups. Descriptive statistics were used to summarize participant characteristics, including sex, age, height, and weight, and are reported as means and standard deviations. The normality of all dependent variables was assessed using the Kolmogorov–Smirnov test to guide the selection of parametric or non-parametric statistical analyses.

Depending on data distribution, non-parametric tests (Wilcoxon signed-rank test and Mann–Whitney U test) and parametric tests (paired- and independent-samples t-tests) were employed to examine within-group and between-group differences in cognitive, emotional, and gross motor outcomes before and after the intervention. Given the exploratory and practice-based nature of the study and the relatively small sample size, no a priori sample size calculation was conducted.

To support interpretation beyond statistical significance, effect sizes and corresponding 95% confidence intervals were reported for all primary outcomes. Effect sizes were calculated using Cohen’s d for parametric analyses and rank-based effect size (r = Z/√N) for nonparametric analyses. Cohen’s d values of 0.2, 0.5, and 0.8 were interpreted as small, medium, and large effects, respectively [22], whereas r values of 0.1, 0.3, and 0.5 were interpreted as small, medium, and large effects. In addition, age-based subgroup analyses were conducted to explore potential developmental differences; however, these analyses further reduced the effective sample size within each subgroup and were therefore interpreted with appropriate caution.

In exploratory, practice-based research conducted in real-world early childhood education settings, formal power analyses and more complex repeated-measures statistical models are often constrained by participant availability and organizational structure. Accordingly, the present study prioritized feasibility and ecological validity over statistical optimization, and the findings are intended to inform preliminary effect estimation and hypothesis generation rather than to provide definitive tests of intervention effectiveness.

All statistical analyses were performed using IBM SPSS Statistics (version 22.0). The significance level was set at *p* < 0.05.

## 3. Results

### 3.1. Cognition Performance Results

At baseline, no significant differences were observed between the experimental and control groups in attention, and number sense in the 4–5-year-old and 5–6-year-old subgroups (all *p* > 0.05) (Table 3). Following the intervention, a significant within-group improvement in working memory was observed in the experimental group (*p* = 0.026, d = 0.84, 95% CI [0.24, 2.95]) in the 5–6-year-old. In addition, gain scores for working memory were significantly greater in the experimental group than in the control group (*p* = 0.04, r = 0.421). No significant within-group or between-group differences were observed for number sense across age groups.

Attention gain scores showed significant within-group improvements in the experimental group for both the 4–5-year-old (*p* = 0.032, d = 0.86, 95% CI [1.35, 23.75]) and 5–6-year-old subgroups (*p* = 0.004, d = 1.22, 95% CI [5.38, 20.61]). No significant pre–post changes in attention were observed in the control group across either age subgroup (all *p* > 0.05). No significant within-group or between-group differences were observed for number sense in either age subgroup (all *p* > 0.05).

### 3.2. Emotional Development Results

Emotional development outcomes are presented in Table 4. At baseline, no significant differences were observed between the experimental and control groups in emotional competence for either age subgroup (all *p* > 0.05). Following the intervention, significant between-group differences were observed in the 4–5-year-old subgroup for total emotional capacity (*p* = 0.013, d = 1.75, 95% CI [7.19, 49.96]), understanding one’s own emotions (*p* = 0.031, d = 1.15, 95% CI [0.58, 4.27]), and emotional adjustment (*p* = 0.035, d = 1.35, 95% CI [0.55, 7.89]), with higher scores in the experimental group.

In the 5–6-year-old subgroup, a significant between-group difference was observed only for understanding others’ emotions (*p* = 0.021, d = 1.32, 95% CI [0.64, 10.58]). Although several emotional capacity domains demonstrated statistically significant effects, the small subgroup sample sizes and multiple outcome comparisons warrant cautious interpretation.

### 3.3. Body Motor Development Results

Body motor development outcomes are presented in Table 5. No significant baseline differences were observed in the 4–5-year-old subgroup (*p* > 0.05). In the 5–6-year-old subgroup, object manipulation scores were higher in the experimental group at baseline; therefore, post-intervention results were interpreted with caution.

In the 4–5-year-old experimental group, significant within-group improvements were observed after the intervention for total body movement (*p* = 0.001, d = 1.65, 95% CI [5.9, 16.1]), locomotion (*p* < 0.001, d = 1.92, 95% CI [4.13, 9.64]), and object manipulation (*p* = 0.02, d = 1.57, 95% CI [1.98, 5.78]). Gain score analyses indicated that improvements in total body movement (*p* = 0.031, d = 1.19, 95% CI [5.9, 14.4]) and object manipulation (*p* = 0.042, d = 0.89, 95% CI [0.82, 3.43]) were significantly greater in the experimental group than in the control group.

In the 5–6-year-old subgroup, no significant between-group differences were observed post-intervention; however, within-group improvements were noted in the experimental group for total body movement (*p* = 0.028, r = 0.54) and object manipulation (*p* = 0.04, r = 0.428).

## 4. Discussion

According to the WHO global physical activity guidelines, children and adolescents should accumulate an average of at least 60 min per day of moderate-to-vigorous intensity physical activity, most of which should be aerobic in nature, to confer substantial health benefits [23]. However, in real-world educational practice, there remains a need for structured, safe, and developmentally appropriate physical activity approaches that can simultaneously support physical, cognitive, and emotional development in young children. To address this gap, the present study designed a structured physical activity program grounded in fundamental movement skills and examined its associations with physical, cognitive, and emotional outcomes. The findings suggest that developmental responses to the program varied across age groups, particularly between children aged 4–5 and those aged 5–6 years. From an applied perspective, the program may provide a practical reference for preschool teachers and childcare providers seeking to implement developmentally appropriate physical activity interventions, while recognizing the contextual constraints of educational settings. In doing so, the study extends existing literature by examining age-specific developmental responses to a structured, school-based physical activity program in a real-world preschool context.

The differential patterns observed between the 4–5-year-old and 5–6-year-old groups highlight the importance of developmental stage in moderating responsiveness to structured physical activity interventions. Younger preschool children may be particularly sensitive to interventions targeting fundamental motor and object manipulation skills, given rapid maturation in basic motor coordination during this period. In contrast, older preschool children may demonstrate greater gains in cognitive domains such as attention and working memory, as executive functions become more differentiated and stable with age. These findings suggest that the same physical activity program may support distinct developmental outcomes depending on children’s developmental readiness.

From a physical development perspective, the intervention emphasized fundamental movement skills through ball-based activities using a variety of equipment, including yoga balls, bubble balls, and rubber balls. Following the intervention, greater gains in object manipulation skills and overall body movement were observed among children aged 4–5 years. Similar patterns have been reported in prior intervention studies demonstrating that structured ball-related activities can support the development of object control skills and overall motor competence in preschool-aged children, such as improvements observed following table tennis or soccer-based programs in children aged 5–7 years (e.g., [24]). These findings suggest that exposure to diverse ball-handling tasks may offer varied motor affordances that support early motor development. Collectively, the present findings align with prior evidence indicating that structured, skill-oriented physical activity programs may support motor development under ecologically valid preschool conditions.

With respect to emotional development, previous studies have reported associations between regular participation in physical activity and improvements in emotional regulation, social engagement, and psychological well-being among young children [24,25,26,27]. In the present study, improvements were observed in selected domains of emotional competence, including emotional understanding and adjustment. These outcomes may be related to the curriculum’s use of thematic activities, cooperative games, and mixed-age interactions, which provided social contexts conducive to emotional learning. However, given methodological constraints, these findings should be interpreted as indicative rather than definitive, and further research is required to clarify the mechanisms linking physical activity participation to emotional development in early childhood.

Evidence regarding the relationship between physical activity and cognitive outcomes in young children remains mixed. A systematic review by Stillman et al. (2020) [28] emphasized that current evidence is limited and inconclusive, particularly for preschool-aged populations, highlighting the need for more rigorously designed trials. Some intervention studies have reported improvements in attention and working memory following structured physical activity involving coordination, balance, and ball skills [4,29], whereas others suggest that cognitive benefits may depend on activity intensity, task complexity, and recovery periods [30,31]. The present study contributes to this literature by suggesting that regular participation in a structured physical activity program may be associated with improvements in attention and working memory, particularly among older preschool children. Importantly, the lack of observed improvements in number sense aligns with prior findings indicating that early numeracy development may be more strongly influenced by direct instructional and counting-based activities than by physical activity alone [32,33,34,35].

Although the present findings suggest associations between participation in a structured physical activity program and improvements in selected developmental outcomes, several methodological considerations warrant explicit attention. Given the quasi-experimental design, small sample size, age-based subgroup analyses, and absence of statistical adjustment for baseline differences, causal inferences cannot be established. Rather, the results should be interpreted as exploratory and hypothesis-generating, consistent with prior applied research in early childhood settings where ecological validity is often prioritized over experimental control [28]. The transferability of the present findings to other cultural contexts should also be considered with caution. Early childhood education systems vary substantially across countries in curriculum structure, pedagogical practices, teacher training, and parental expectations regarding physical activity. Cross-cultural research has demonstrated that children’s motor competence, number sense, and cognitive engagement differ across educational systems due to sociocultural and instructional factors [34,36]. Accordingly, the feasibility and potential benefits of the proposed physical activity program may vary when implemented in educational contexts with differing curricular priorities or resource availability.

Furthermore, the real-world applicability of the intervention warrants careful reflection. The present study implemented a relatively high frequency of physical activity sessions, which may not be feasible in many preschool or childcare settings due to time constraints, staffing limitations, or curricular demands. Prior research suggests that lower-frequency or shorter-duration physical activity interventions may yield more modest or domain-specific benefits, particularly for cognitive outcomes [37]. Consequently, the observed effects may not directly generalize to settings where physical activity is delivered less frequently. Accordingly, future research should therefore explore the minimal effective dose of structured physical activity and examine whether comparable developmental benefits can be achieved under more constrained, real-world implementation conditions.

## 5. Limitations

Several limitations of the present study should be acknowledged. The relatively small sample size, partly attributable to declining birth rates and the substantial instructional and personnel resources required to implement the newly developed physical activity curriculum, may limit the generalizability of the findings and constrain statistical power, particularly in age-based subgroup analyses. The relatively short duration of the intervention may have limited the detection of longer-term or sustained developmental effects. In addition, individual differences in children’s prior physical activity levels were not fully controlled, and contextual factors such as family environment and home-based cognitive stimulation were not systematically assessed, potentially contributing to variability in responsiveness to the intervention. From a measurement and intervention perspective, the adapted cognitive assessment tasks lacked established psychometric properties, and their sensitivity to detect short-term changes over the 8-week intervention period has not been formally validated. Furthermore, physical activity intensity was not objectively quantified, and implementation fidelity was not formally assessed; despite the use of a structured curriculum and a single instructor, these factors may limit precise characterization of intervention exposure and dose–response relationships. Collectively, these limitations may have introduced potential sources of bias; therefore, the findings should be interpreted with appropriate caution. Although randomized controlled designs are methodologically stronger, their implementation in early childhood educational settings is often constrained by ethical, administrative, and organizational considerations; accordingly, a quasi-experimental design was adopted to balance methodological rigor with ecological validity. Future studies employing larger and more diverse samples, extended intervention periods, multi-site designs, and more comprehensive assessments of baseline activity levels, intervention intensity, implementation fidelity, psychometrically validated cognitive measures, and family-related contextual factors are warranted to validate and extend the present findings. Accordingly, the present findings should be interpreted as preliminary, and future studies employing adequately powered samples and repeated-measures statistical models (e.g., mixed-effects models or MANOVA) are warranted to confirm and extend these results.

## 6. Practical Implications

The present study provides several practical implications for interdisciplinary research and educational practice in early childhood settings. From an educational perspective, the findings suggest that structured and developmentally appropriate physical activity programs can be feasibly incorporated into early childhood curricula to support multiple domains of child development, including attention, emotional regulation, and motor skills. This has practical relevance for educators seeking evidence-based strategies that align with both developmental needs and classroom realities.

From an intervention design perspective, the implementation of a newly developed physical activity curriculum in this study offers a practical reference for practitioners and program developers. The results indicate that even relatively short-term, well-structured physical activity interventions may yield measurable developmental benefits, which is particularly important in educational contexts where time and human resources are limited.

In addition, the findings contribute to interdisciplinary research by bridging concepts from physical activity science, developmental psychology, and early childhood education. For researchers, the study underscores the importance of adopting applied, school-based designs when examining physical activity interventions in young children and highlights the need for future studies to explore age-specific responses, longer intervention durations, and real-world implementation factors.

Overall, these practical implications support the integration of physical activity–based approaches as accessible and scalable strategies within early childhood education and provide direction for future interdisciplinary research focused on child development.

## 7. Conclusions

This study examined the effects of an 8-week programmed physical activity (PA) intervention on cognitive, emotional, and motor development in children aged 4–6 years using a quasi-experimental design. The findings suggest that participation in the structured PA program was associated with improvements in selected domains of cognitive performance (particularly attention and working memory), emotional capacity, and motor development, with patterns varying across age subgroups. Given the exploratory nature of the study, the relatively small sample size, age-based subgroup analyses, and the absence of statistical adjustment for baseline differences, the findings should be interpreted with appropriate caution. Rather than providing definitive causal evidence, the present results offer preliminary support for the potential role of structured PA interventions in early childhood developmental contexts.

From an applied perspective, the study provides ecologically valid insights into the feasibility of implementing structured PA programs within preschool settings. These findings may inform the design of future educational and interdisciplinary research employing larger samples, longer intervention periods, and more rigorous methodological controls to further examine the developmental benefits of physical activity in young children.

## Figures and Tables

**Table 1:** Control variables of the participants.

		Experimental Group			Control Group			
		*n*	Mean		*n*		Mean	
			Height (cm)	Weight (kg)			Height (cm)	Weight (kg)
4–5 years	Male	3	104 ± 6.3	16.0 ± 1.8	Male	3	106 ± 5.8	17.3 ± 1.9
	Female	6	106 ± 4.8	17.4 ± 2.9	Female	2	107 ± 0.1	16.5 ± 1.3
5–6 years	Male	6	110 ± 3.4	16.5 ± 1.3	Male	6	114 ± 3.6	18.6 ± 1.9
	Female	4	110 ± 4.5	18.2 ± 2.7	Female	3	109 ± 4.5	16.5 ± 1.3

**Table 2:** 8-week programmed physical activity intervention.

Week	Theme	Fundamental Movement Skills *	Programmed Physical Activity Intervention
1	Soccer prince	Stability (Stretch, Bend, Rotate) Locomotor (Walk, Run) Object control (Kick, Strike)	(1) Sandbag balance (2) Kicking expert (3) Shooting expert (4) Bowling
2	Nice pitcher	Stability (Squat, Rotate, Swing) Locomotor (Walk, Run, Jump) Object control (Throw, Strike, dribble)	(1) Walking with frisbee on top of the head (2) Launching a frisbee (3) Frisbee shooting (4) Soccer frisbee (5) Dodging a frisbee
3	Shooting expert	Stability (Bend, Squat, Swing) Locomotor (Walk, Step, Slide) Object control (Throw, Catch, Dribble)	(1) Dunking and turning (2) Walking with the ball (3) Dribbling practice (4) Rolling and catching (5) Catching with a basket (6) Shooting and passing
4	Hula hooping	Stability (Stretch, Bend, Squat, Swing) Locomotor (Walk, Run, Jump, Slide) Object control (Catch, Strike)	(1) Hula hoop running (2) Hula hoop skipping (3) Night market fishing and tire rolling (4) Crab-walking and kangaroo hopping (5) Musical note hula hoop—big bad wolf and little pigs (6) Castle assault (7) Happy farm
5	Mini-cone supporting	Stability (Bend, Squat, Swing) Locomotor (Walk, Run, Jump, Slide) Object control (Throw)	(1) Running around the cones (2) Corne kicking and ring toss (3) Cone kick and ring toss (4) Tripping and supporting (5) Detective task 1—plastic ball search (6) Detective task 2—identical colored ball search
6	Inflatable air Stick	Stability (Bend, Squat, Swing) Locomotor (Run, Step, Jump, Slide) Object control (Strike)	(1) High jump and hitting, crab-walking together (2) Wolf’s tail, whac-a-mole (3) High jump and long jump, small hotdog (4) Air stick rescue (5) Walking with an air stick
7	Bobble training	Stability (Bend, Squat, Rotate, Swing) Locomotor (Walk, Run) Object control (Throw, Catch)	(1) Diamond game (2) Pass and shooting (3) Turning, passing, catching, and shooting (4) Bobble rain, egg-catching game
8	Pushing a yoga ball	Stability (Stretch, Squat, Rotate) Locomotor (Walk, Run, Jump) Object control (Throw, Kick)	(1) Dodging a yoga ball (2) Rock attack (3) Dinosaur egg game (4) Turning in circles to dodge a ball

* Fundamental movement skills include locomotor skills, object control skills, and stability skills.

**Table 3:** Effects of the 8-week programmed physical activity intervention on the cognitive development of the young children.

	4–5-Year-Old Group					
					Gain Score	
	Experimental Group		Control Group		Experimental Group	Control Group
	**Pre-Test**	**Post-Test**	**Pre-Test**	**Post-Test**		
Working memory	5.89 ± 2	6.00 ± 2	7.00 ± 2	7.20 ± 1	1.11 ± 2	0.20 ± 1
Number sense	9.11 ± 1	8.67 ± 1	9.20 ± 1	9.60 ± 1	−0.44 ± 1	0.40 ± 1
Attention	28.33 ± 7	40.89 ± 11 *	32.40 ± 13	39.00 ± 6	12.56 ± 14	6.60 ± 9
	**5–6-Year-Old Group**					
					**Gain Score**	
	**Experimental Group**		**Control Group**		**Experimental Group**	**Control Group**
	**Pre-Test**	**Post-Test**	**Pre-Test**	**Post-Test**		
Working memory	4.90 ± 2 ^$^	6.50 ± 1 *	7.00 ± 3	6.78 ± 1	1.60 ± 2 ^#^	−0.22 ± 2
Number sense	9.20 ± 1	9.10 ± 1	9.44 ± 1	9.56 ± 1	−0.10 ± 1	0.11 ± 1
Attention	35.50 ± 13	48.50 ± 13 *	41.67 ± 17	50.22 ± 11	13.00 ± 11	8.56 ± 16

Data are presented as mean ± standard deviation (SD). * Indicates within-group comparisons using paired-samples *t* tests. $ Indicates between-group comparisons of pre-test scores using independent-samples *t* tests, and # indicates between-group comparisons of post-test scores and gain scores (post-test minus pre-test) using independent-samples *t* tests. Exact *p* values, effect sizes, and 95% confidence intervals are reported in Section 3.1.

**Table 4:** Effects of the 8-week programmed physical activity intervention on the emotional development of the young children.

	4–5-Year-Old Group					
					Gain Score	
	Experimental Group		Control Group		Experimental Group	Control Group
	**Pre-Test**	**Post-Test**	**Pre-Test**	**Post-Test**		
Total emotion score	110.33 ± 24	162.78 ± 19 *^#^	129.00 ± 10	134.20 ± 11	52.44 ± 10 ^#^	5.20 ± 4
Understanding one’s emotions	28.22 ± 8	42.44 ± 5 *^#^	32.60 ± 4	34.40 ± 3	14.22 ± 6 ^#^	1.80 ± 1
Understanding others’ emotions	28.11 ± 7	40.56 ± 7 *	35.80 ± 4	36.80 ± 4	12.44 ± 5 ^#^	1.00 ± 1
Emotional adjustment	28.44 ± 7	42.67 ± 4 *^#^	28.20 ± 1	28.80 ± 2	14.22 ± 3 ^#^	0.60 ± 1
Self-motivation	25.56 ± 7	37.11 ± 5 *	32.40 ± 4	34.20 ± 4	11.56 ± 4 ^#^	1.80 ± 1
	**5–6-Year-Old Group**					
					**Gain Score**	
	**Experimental Group**		**Control Group**		**Experimental Group**	**Control Group**
	**Pre-Test**	**Post-Test**	**Pre-Test**	**Post-Test**		
Total emotion score	111.00 ± 25	173.90 ± 15 *	136.56 ± 27	153.22 ± 27 *	62.90 ± 14 ^#^	16.67 ± 4
Understanding one’s emotions	30.40 ± 6	44.10 ± 4 *	34.33 ± 7	38.78 ± 7 *	13.70 ± 4 ^#^	4.44 ± 3
Understanding others’ emotions	28.00 ± 7	45.30 ± 4 *^#^	33.78 ± 6	38.78 ± 7 *	17.30 ± 7 ^#^	5.00 ± 2
Emotional adjustment	26.00 ± 7 ^$^	42.80 ± 6 *	34.00 ± 6	38.89 ± 7 *	16.20 ± 4 ^#^	4.89 ± 2
Self-motivation	26.00 ± 5 ^$^	41.70 ± 4 *	34.44 ± 6	36.78 ± 6 *	15.70 ± 3 ^#^	2.33 ± 3

Data are presented as mean ± SD. * Indicates within-group comparisons using paired-samples *t* tests. $ Indicates between-group comparisons of pre-test scores using independent-samples *t* tests and # indicates between-group comparisons of post-test scores and gain scores (post-test minus pre-test) using independent-samples *t* tests. Exact *p* values, effect sizes, and 95% confidence intervals are reported in Section 3.2.

**Table 5:** Effects of the 8-week programmed physical activity intervention on the young children’ body motor development.

	4–5-Year-Old Group					
					Gain Score	
	Experimental Group		Control Group		Experimental Group	Control Group
	**Pre-Test**	**Post-Test**	**Pre-Test**	**Post-Test**		
Total body movement score	64.44 ± 9	75.44 ± 8 *	67.80 ± 3	64.40 ± 14	11.00 ± 6 ^#^	−3.40 ± 15
Locomotion	31.22 ± 5	38.11 ± 3 *	33.80 ± 7	32.40 ± 8	6.89 ± 3	−1.80 ± 8
Object manipulation	17.78 ± 2	21.67 ± 1 *	18.00 ± 2	17.80 ± 5	3.89 ± 2 ^#^	−0.20 ± 3
Balance	15.44 ± 2	15.67 ± 3	15.60 ± 1	14.20 ± 3	0.22 ± 2	−1.40 ± 5
	**5–6-Year-Old Group**					
					**Gain Score**	
	**Experimental Group**		**Control Group**		**Experimental Group**	**Control Group**
	**Pre-Test**	**Post-Test**	**Pre-Test**	**Post-Test**		
Total body movement score	72.80 ± 14	79.00 ± 7 *	71.22 ± 5	73.78 ± 6	6.20 ± 8	2.56 ± 8
Locomotion	36.50 ± 5	36.7 ± 3	37.22 ± 2	37.44 ± 4	3.00 ± 3	0.22 ± 4
Object manipulation	19.8 ± 3 ^$^	22.60 ± 4 *	16.78 ± 3	19.67 ± 3	1.80 ± 1	2.89 ± 5
Balance	15.90 ± 5	17.30 ± 1	17.22 ± 1	17.33 ± 1	1.40 ± 5	0.11 ± 1

Data are presented as mean ± SD. * Indicates within-group comparisons using paired-samples *t* tests. $ Indicates between-group comparisons of pre-test scores using independent-samples *t* tests and # indicates between-group comparisons of post-test scores and gain scores (post-test minus pre-test) using independent-samples *t* tests. Exact *p* values, effect sizes, and 95% confidence intervals are reported in Section 3.3.

## Data Availability

The datasets used and analyzed in this study are available from the corresponding author upon reasonable request.

## Abbreviations

The following abbreviations are used in this manuscript:

FMS	Fundamental movement skills
PA	Physical activity
PGMQS	Preschooler Gross Motor Quality Scale
ADHD	Attention-deficit/hyperactivity disorder
